# Histopathology of COVID-19: An illustration of the findings from fatal cases

**DOI:** 10.7705/biomedica.6737

**Published:** 2023-03-30

**Authors:** Jorge Rivera, Sheryll Corchuelo, Julián Naizque, Édgar Parra, Eugenio Aladino Meek, Diego Álvarez-Díaz, Marcela Mercado, Orlando Torres-Fernández

**Affiliations:** 1 Grupo de Morfología Celular, Dirección de Investigación en Salud Pública, Instituto Nacional de Salud, Bogotá, D.C., Colombia Dirección de Investigación en Salud Pública Instituto Nacional de Salud Bogotá, D.C. Colombia; 2 Grupo de Patología , Dirección de Redes en Salud Pública, Instituto Nacional de Salud, Bogotá, D.C., Colombia Dirección de Redes en Salud Pública Instituto Nacional de Salud Bogotá, D.C. Colombia; 3 Genómica de Microorganismos Emergentes, Dirección de Investigación en Salud Pública, Instituto Nacional de Salud, Bogotá, D.C., Colombia Dirección de Investigación en Salud Pública Instituto Nacional de Salud Bogotá, D.C. Colombia; 4 Dirección de Investigación en Salud Pública, Instituto Nacional de Salud, Bogotá, D.C., Colombia Dirección de Investigación en Salud Pública Instituto Nacional de Salud Bogotá, D.C. Colombia

## Commentary

COVID-19 represents the greatest global public health crisis since the influenza pandemic of 1918 ^1^. Since its first report in December, 2019, the SARS-CoV-2 coronavirus responsible for coronavirus disease 2019 (COVID-19) has efficiently transmitted from person to person, and two years after the declaration of the pandemic by the World Health Organization (WHO), it has caused approximately 481,756,671 infections and 6,127,981 deaths worldwide ^2^.

All age groups are affected by COVID-19 without excluding any condition, including pregnant women and children. Older people are more predisposed to the development of the disease, and patients with comorbidities such as obesity, diabetes, hypertension, and cardiovascular disease often have a worse prognosis ^3^^,^^4^. The disease presents a clinical picture in a range that can vary between an asymptomatic condition to multiorgan failure and death ^5^. In adults, it usually manifests as fever, cough, and fatigue, and in some cases, it is accompanied by nasal discharge, headache, and other infrequent symptoms, such as diarrhea and gastrointestinal disorders ^6^.

Coronaviruses are viruses that belong to the Coronavirinae subfamily in the Coronaviridae family and can cause disease in both animals and humans ^7^. The viral particle, which resembles a crown in electron micrographs, has a size that varies between 80 and 220 nm and carries the most extensive genome of positive-strand RNA viruses; this genome contains five essential genes, of which four encode structural proteins (N, E, M and S) and one encodes a protein for transcription/replication (RNA-dependent RNA polymerase, RdRp). The organization in the genome is 5’-RdRp-S-E-M-N-3’, and this order is highly conserved among coronaviruses ^7^.

The SARS-CoV virus enters its host cell through the binding of the S protein to the ACE2 (angiotensin-converting enzyme 2) receptor; this binding determines the tropism of the virus and viral pathogenesis ^8^^,^^9^. Infection with SARS-CoV mainly generates pneumonia-like symptoms, and the lung is the most pathologically affected organ ^10^^,^^11^. Studies on the histopathology of COVID-19 have reported macroscopic findings such as pleurisy, pericarditis, consolidation, edema and pulmonary hemorrhage, cardiomegaly, and ventricular dilation ^12^^,^^13^.

In the first descriptions of the histopathological findings of SARS- CoV-2, diffuse alveolar damage, hyaline membrane formation and vascular congestion, hemorrhage and fibrinoid deposits in the intra-alveolar space, and inflammatory mononuclear cell infiltration, edema, interstitial fibrosis, and hypertrophy in the myocardium were observed ^14^. Additionally, pulmonary lesions, such as the desquamation of pneumocytes and the presence of hyaline membranes and edema, have been observed, signs of acute respiratory distress syndrome. Tracheobronchitis with mononuclear cell inflammation, epithelial denudation and submucosal congestion, alveolar infiltrate with alveolar macrophage hyperplasia and mononuclear inflammatory interstitial infiltrate have also been observed ^15^^,^^16^. These alterations are not exclusive to SARS-CoV-2. There are predominant histopathological patterns, such as diffuse alveolar damage, that are shared with other respiratory viruses, such as SARS and influenza; however, vascular alterations, such as thrombosis and microthrombosis, seem to be more frequent in cases of COVID-19 and SARS, which suggests that coronaviruses in general could be associated with an increase in pulmonary microthrombi ^17^.

Other organs that have presented histological alterations associated with SARS-CoV-2 infection are the liver, kidney and heart. For example, in the liver, cirrhosis, moderate microvesicular steatosis and mild portal and lobular activity have been observed; these alterations can be associated with viral infection or drug-induced damage. In the kidney, chronic kidney disease and acute duct lesions have been described, and in the heart, myocardial fibrosis and mild mononuclear inflammatory infiltrate have been observed ^3^.

In Colombia, due to biosecurity issues in the framework of the health emergency due to COVID-19, the routine execution of necropsies, viscerotomies and *postmortem* tissue sampling by invasive methods was restricted; therefore, there is little material from tissues of infected patients as sources of useful information to understand the pathogenesis of the disease and thus few studies on the histopathology of viral infection by SARS-CoV-2. However, among some fatal cases in the *Red Nacional de Laboratorios* that were initially associated with mortality due to non-COVID acute respiratory infection that underwent routine necropsy, SARS-CoV-2 infection was confirmed by differential laboratory diagnosis, *postmortem* or some time before death. The tissue samples obtained in these cases are in the archives of the *Laboratorio de Patología* of the *Instituto Nacional de Salud*.

As a contribution to the understanding of the pathogenesis of COVID-19 and the morphological alterations caused by infection with SARS-CoV-2, this study illustrates the histopathological alterations and their frequencies among a group of 50 fatal cases of COVID-19 in Colombia (figures 1-11). The histopathological characterization of the cases was performed using histological preparations with routine hematoxylin and eosin staining. 

Lung

**Figure 1:**
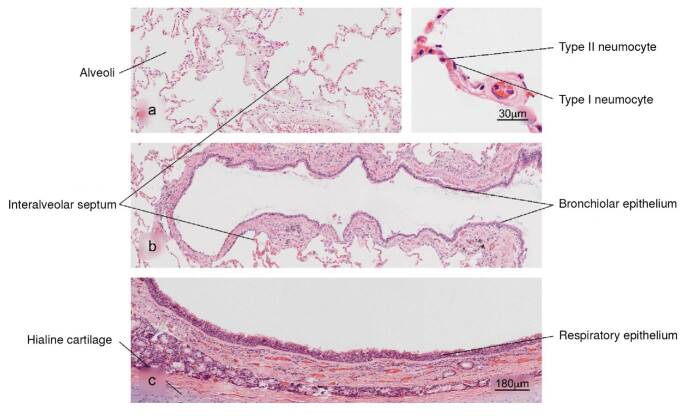
Normal histology of the lung. a) Alveolar region; note the completely free alveoli for oxygen supply through the capillaries located in the interalveolar septa, b) bronchiole in longitudinal section and c) trachea - upper respiratory tract. H&E stain.

**Figure 2:**
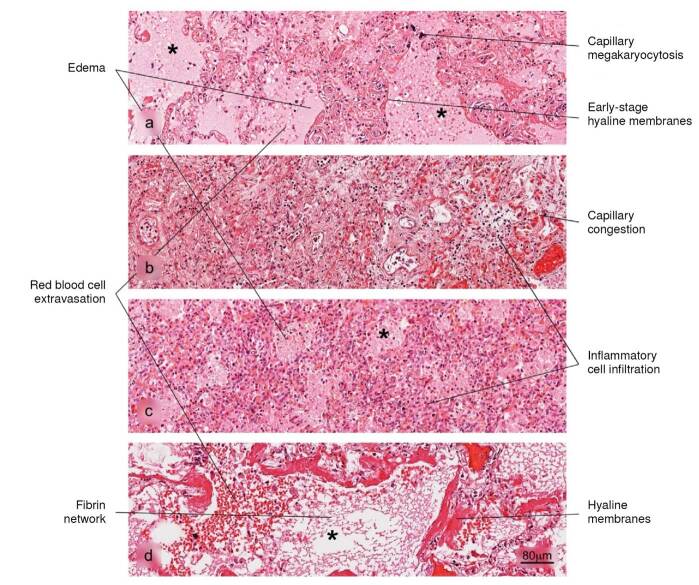
Histopathological alterations in lung tissue associated with COVID-19. The different phases of diffuse alveolar damage (DAD) observed in fatal cases are illustrated: a) acute, b) acute proliferative, c) proliferative and d) fibrotic. Pulmonary alveolus (*). H&E stain.

**Figure 3:**
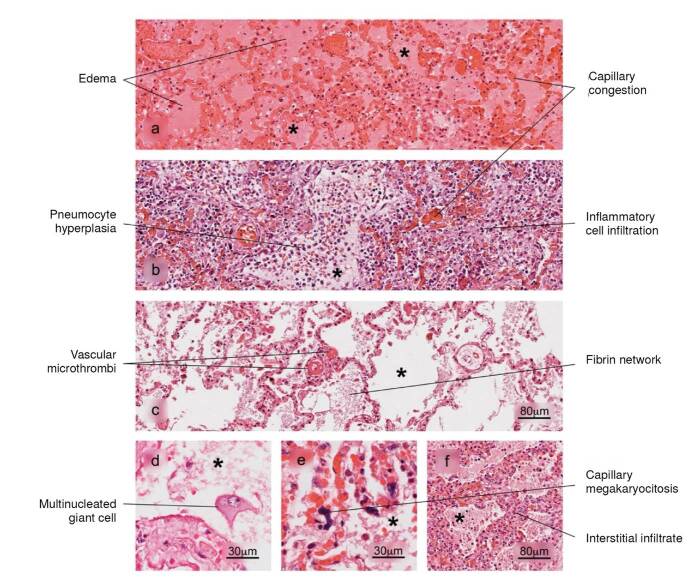
Histopathological alterations in lung tissue associated with COVID-19. a) edema, characterized by the presence of protein content in the alveolar regions, b) reactive pneumocyte hyperplasia, c) vascular microthrombi, d) presence of multinucleated giant cells, e) capillary megakaryocytosis and f) lymphocytic interstitial infiltrate. Pulmonary alveolus (*). H&E stain.

**Figure 4:**
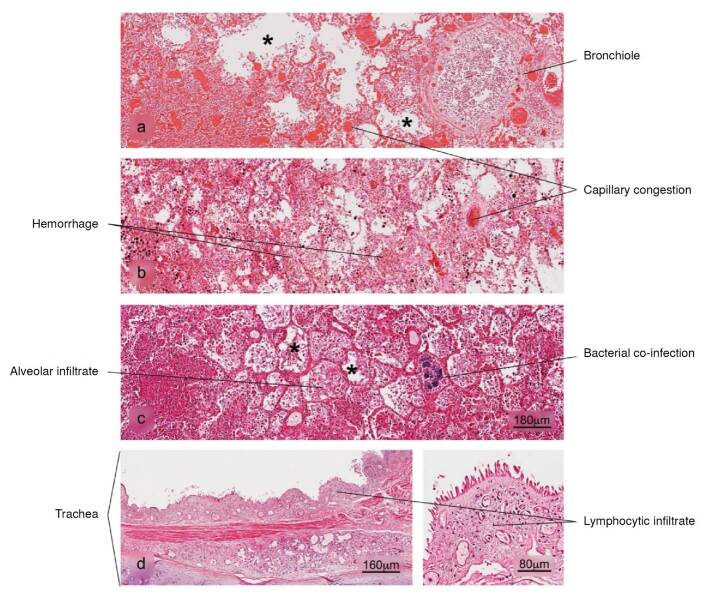
Histopathological alterations in lung tissue associated with COVID-19. a) bronchopneumonia, b) hemorrhage, c) superaggregated lobar pneumonia, d) inflammation of the trachea. Pulmonary alveolus (*). H&E stain.

**Figure 5:**
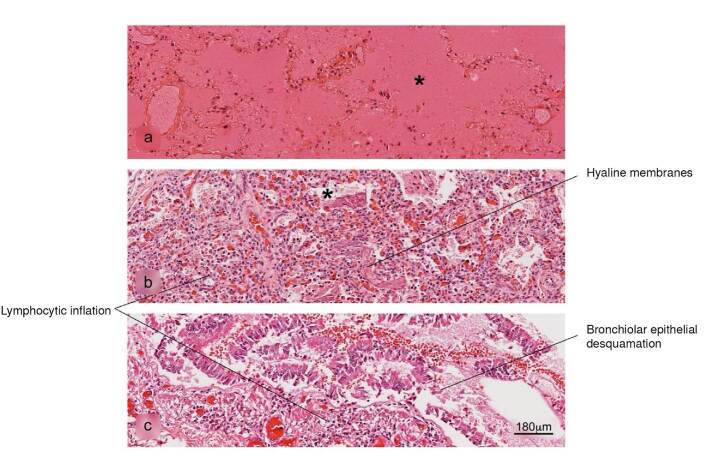
Histopathological alterations in lung tissue associated with COVID-19. a) pulmonary infarction, b) acute pneumonitis with fibrinoid exudate, c) bronchiolar inflammation. Pulmonary alveolus (*). H&E stain.

Spleen

**Figure 6:**
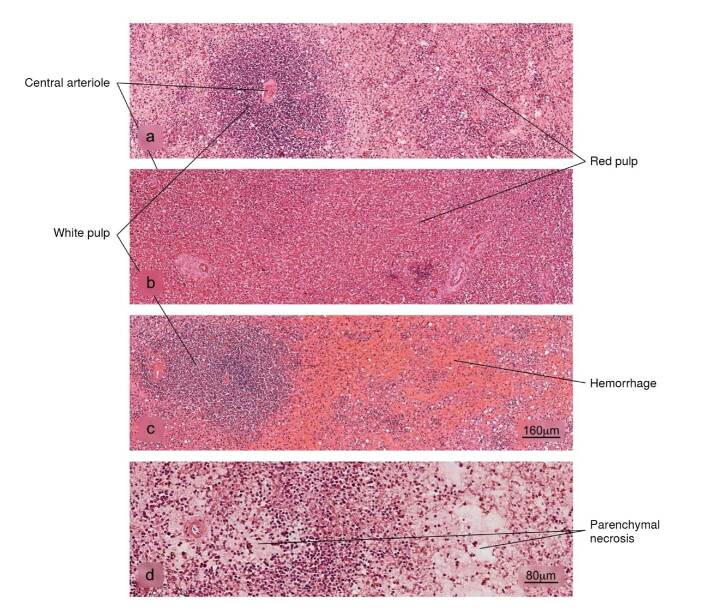
Histopathological alterations in splenic tissue associated with COVID-19. a) Normal histology of the spleen in which a lymphatic node - white pulp - and an extensive area of red pulp is observed, b) reduction in the white pulp, c) hemorrhage in the red pulp, d) parenchymal necrosis. H&E stain.

**Figure 7:**
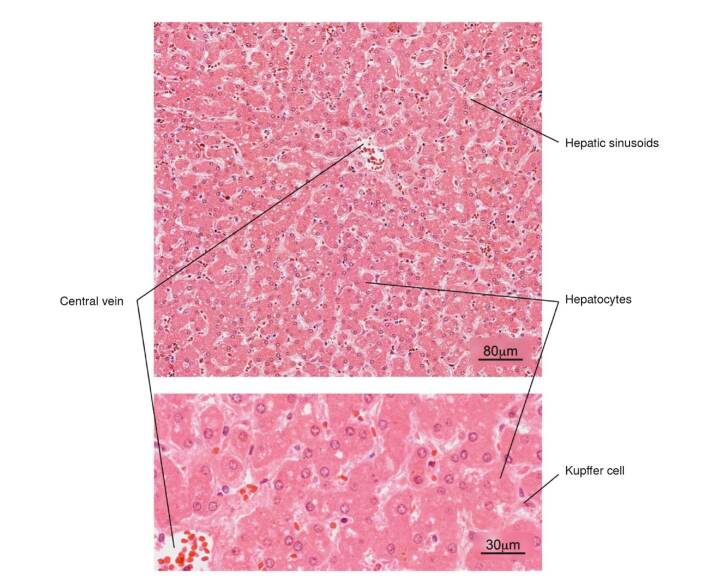
Normal histology of the liver. Note the radial arrangement of the hepatic plates from the central vein of a hepatic lobule. The lower image shows in more detail the hepatocytes and some Kupffer cells located in the sinusoids. H&E stain.

Liver

**Figure 8:**
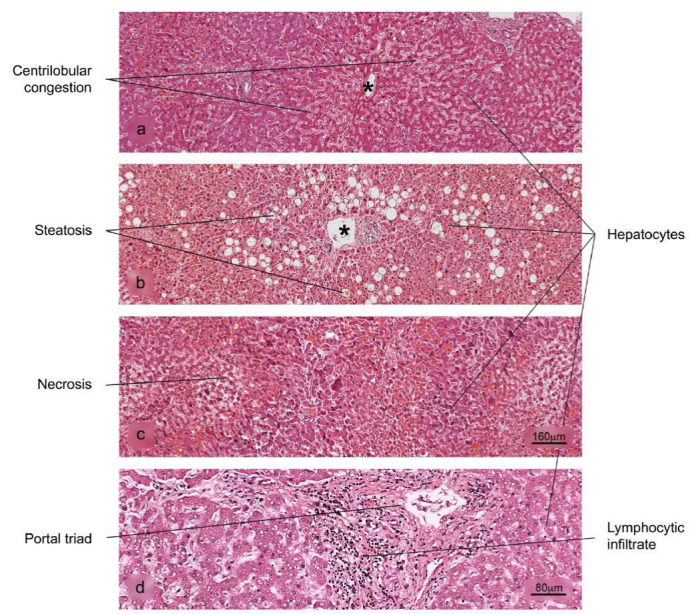
Histopathological alterations in liver tissue associated with COVID-19. a) sinusoidal congestion, b) fatty liver degeneration - macro- and microvesicular steatosis, c) necrosis, d) lymphocytic infiltrate of the portal triad. H&E stain.

Kidney

**Figure 9:**
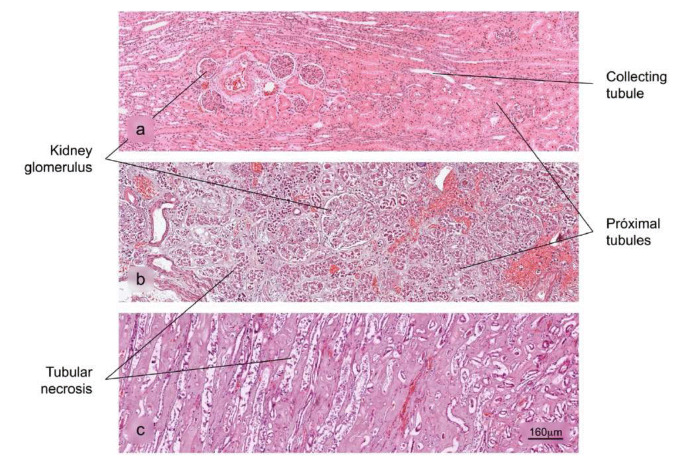
Histopathological alterations in kidney tissue associated with COVID-19. a) Normal histology of the kidney. Renal corpuscles are observed in the cortex. b) and c) Acute duct necrosis. H&E stain.

Brain

**Figure 10:**
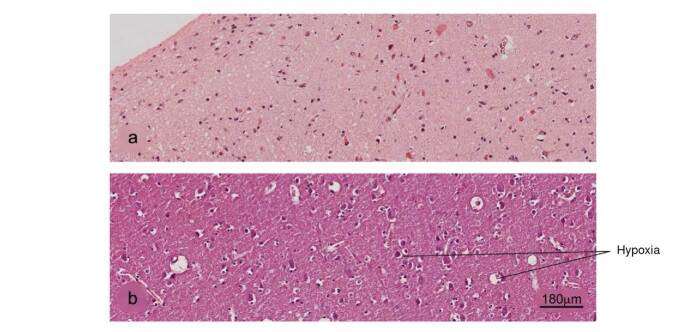
Histopathological alterations in brain tissue associated with COVID-19. a) Normal histology of the cerebral cortex, b) acute hypoxic lesion and c) encephalitis. H&E stain.

**Figure 11:**
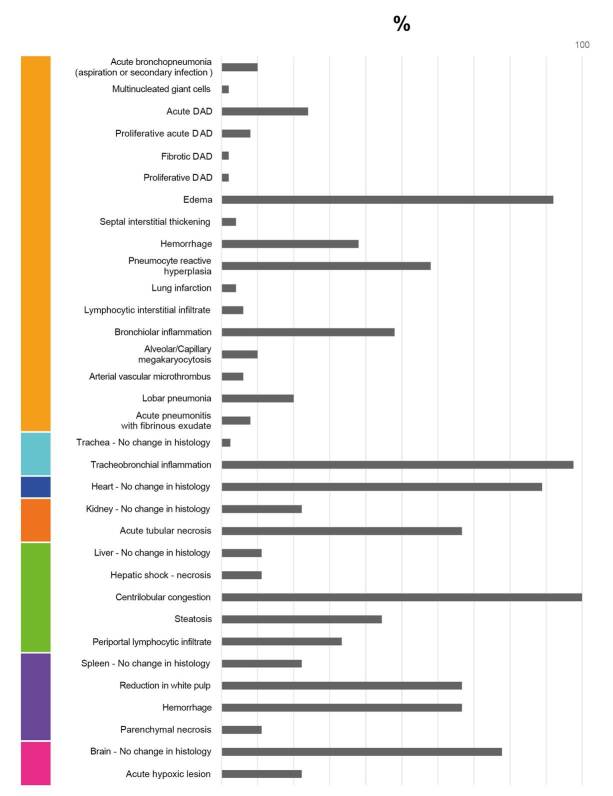
Frequencies of histopathological findings for 50 fatal cases associated with COVID-19.
